# The effect of emergency department occupancy on the revisitation rate within seven days among patients discharged by triage

**DOI:** 10.1186/s12873-025-01315-8

**Published:** 2025-08-15

**Authors:** Jari Ylä-Mattila, Anna Eidstø, Jalmari Nevanlinna, Heini Huhtala, Teemu Koivistoinen, Sami Mustajoki

**Affiliations:** 1 Emergency Department, Tampere University Hospital, P.O. Box 2000, Tampere, FI-33521 Finland; 2 Faculty of Medicine and Health Technology, Tampere University, Tampere, FI-33520 Finland; 3 Faculty of Social Sciences, Tampere University, Tampere, FI-33014 Finland; 4 Emergency Department, Kanta-Häme Central Hospital, Hämeenlinna, FI-13530 Finland; 5 Department of Medicine, Tampere University Hospital, Tampere, FI-33521 Finland

**Keywords:** Crowding, Discharge, Emergency department, Occupancy, Low acuity, Nonurgent, Redirect, Triage

## Abstract

**Background:**

Emergency department (ED) crowding has been repeatedly shown to affect patient outcomes negatively. However, there is limited research on its impact on patients immediately discharged by the triage team. This study aimed to evaluate the effect of ED occupancy level on the rates of ED revisitation and hospitalization within seven days among patients discharged or redirected by the triage team.

**Methods:**

An observational single-center study was conducted at the Tampere University Hospital ED from January 1, 2023, to December 31, 2024. The study population consisted of patients who were discharged or redirected by the ED triage team. These patients were divided into two groups: (1) patients who revisited the ED within seven days and (2) patients who did not return within seven days. A subgroup analysis focused on revisits that resulted in hospitalization. ED occupancy at the time of triage was considered as a predicting factor for revisitation and hospitalization. Age, sex, triage shift, and the updated Charlson Comorbidity Index (uCCI) were adjusted for in a multivariable logistic regression analysis.

**Results:**

Of the 180,267 ED visitors during the study period, 8.8% (*n* = 15,910) were discharged by the triage team. Of these, 8.7% (*n* = 1392) revisited the ED within seven days, and 16.2% (*n* = 225) of the revisiting patients were hospitalized. In the multivariable analyses, the highest quartile of ED occupancy was associated with an increased likelihood of ED revisitation (odds ratio [OR]: 1.29, 95% confidence interval [CI]: 1.06–1.57). Older age was linked to both revisitation and hospitalization (OR for a 1-year increase 1.01 [95% CI: 1.01–1.02] and 1.02 [95% CI: 1.02–1.03], respectively). The uCCI score was also associated with revisitation and hospitalization (OR for a 1-point increase 1.13 [95% CI: 1.07–1.18] and 1.23 [95% CI: 1.13–1.33], respectively).

**Conclusions:**

The highest ED occupancy quartile was associated with a modestly increased likelihood of an ED revisit but not hospitalization within seven days after being discharged by the triage team. Furthermore, age and comorbidities were associated with both revisitation and hospitalization.

**Trial registration:**

Clinical trial number: not applicable.

## Background

The growing demand for emergency department (ED) services has been extensively documented over the years, alongside the associated adverse outcomes resulting from recurring crowding in EDs worldwide [1–3]. Based on the conceptual framework describing the ED crowding mechanisms [4], several interventions have been proposed for reducing ED utilization [1, 5]. The redirecting of nonurgent patients to an appropriate level of care has been explored [6, 7]. Despite several proposals [8–12], there are currently no universally accepted and reliable classifications to determine the appropriate redirection strategy for the nonurgent patient population.

The redirection strategies, however, might have ethical and legal issues, since the ability of triage to predict ED discharge and hospitalization is considered suboptimal and varies between patient groups [13–16]. Furthermore, ED visits classified as nonurgent consist of a heterogenous group of patients seeking ED care [13, 17, 18], and the criteria for ED visits that are considered suitable for redirection vary [8, 9, 11, 12, 19]. Moreover, redirecting nonurgent visits may not enhance the access to necessary acute care for other patients [19], and as a solution for alleviating ED crowding, the evidence of such diversion strategies has been insufficient [1, 20]. In contrast, the triage process is affected by the ED occupancy rate: in prior studies, higher ED occupancy has been associated with longer waiting times for triage [21], with the classification of patients as higher acuity [22], with high-acuity patients being triaged to a non-monitored area [23], and with referrals from a general practitioner back to the ED after being redirected from the ED [21]. However, higher ED occupancy has not been associated with a higher number of patients redirected from the ED [21]. In one study, the permeability of triage even increased when the in-hospital bed occupancy rate was at its highest, and, additionally, high bed occupancy did not increase the rate of ED revisits within 72 h [24].

The adverse outcomes associated with ED crowding are well described [1, 2]. However, the potential solutions, such as the effectiveness of diversion strategies to manage or prevent recurrent crowding, have not been robustly established [1, 20]. Regardless of whether diversion strategies are implemented in the local healthcare system, the relationship between ED occupancy levels and the triage discharge or redirection process has not been thoroughly described. To elucidate this relationship, a retrospective observational study was conducted at the ED of a high-volume tertiary hospital. The aim of this study was to determine the associations of ED occupancy with (1) ED revisits within seven days and with (2) revisits within seven days leading to hospitalization among a group of patients initially directly redirected or discharged by the triage team.

## Methods

### Study design and setting

A retrospective observational study was carried out at Tampere University Hospital in Finland, using data from all ED visits during the years 2023 and 2024. The study was approved by the hospital’s research director (research diary number R24278). The STROBE (Strengthening the Reporting of Observational Studies in Epidemiology) guidelines were applied in this study [25].

Tampere University Hospital provides secondary care for 540,000 residents within the Wellbeing Services County of Pirkanmaa (publicly funded universal healthcare system) and is the only hospital in the region managing all severe emergency situations. Additionally, the hospital functions as a tertiary care unit for a broader area covering 900,000 residents. Each year, the ED handles approximately 90,000 visits and has a total of 65 beds. Additionally, there is a waiting area for walk-in patients who do not require continuous monitoring. The ED serves only adult patients, with the exception of injuries in patients under 16 years of age. There are only a limited number of healthcare units offering services outside regular office hours, and since June 2024, the ED is the only general ED in the region providing nighttime emergency care.

A five-level triage system, the Emergency Severity Index (ESI) [26], is used in the ED. All patients arriving at the ED are assessed by the triage team, with the goal of promptly discharging low-acuity patients or redirecting them to nonurgent healthcare services, such as primary healthcare centers, occupational health services, or private healthcare providers. The ESI classification is not used for directly discharged or redirected patients. The triage team consists of trained nurses, and a physician is always available for consultation either by telephone or, if necessary, being present in the triage situation. The criteria for the interface between emergency and non-emergency care are nationally guided, but the decision on the urgency of care must always be made on a case-by-case basis [27]. The case-by-case evaluation of clinical urgency consists of the patient’s medical history, an interview, an evaluation of the severity of symptoms, and, if appropriate, the measurement of vital signs and point-of-care analyses, including C-reactive protein. Triage nurses are allowed to discharge patients directly or redirect them to other suitable healthcare providers outside the ED without consulting a physician, but they are encouraged to consult a physician in any unclear cases. However, the final decision to discharge or redirect a patient is based on comprehensive clinical evaluation and decision-making according to the national guidance [27], with or without a physician consultation [16].

### Study population and protocol

A retrospective observational study was carried out to examine the relationship between emergency department occupancy levels and the likelihood of revisitation or hospitalization within seven days. The study population consisted of patients who were evaluated and discharged by the triage team. The patients were divided into two groups: (1) those who revisited the ED within seven days after the initial triage discharge or redirection and (2) those who were discharged or redirected and did not return within seven days. In order to manage multiple visits within seven days, each revisit and its outcome (hospitalization or no hospitalization) were recorded independently.

### Study variables

The variables collected from the hospital’s electronic patient records for each patient visit included the personal identity code unique to each permanent Finnish resident, as well as sex, date of birth, age at arrival, the date and time of arrival and discharge, and whether the patient was directly discharged by triage. The actual ED occupancy level at the time of triage discharge was calculated using the arrival and discharge times of patients who were admitted to the ED at the time when the study patient was discharged by triage. Additionally, the triage shift for each of the included visits was defined according to the time of arrival (day 7:30–14:29, evening 14:30–21:59, and night 22:00–7:29). The date of the ED revisit within seven days and data on whether the patient was hospitalized following the revisit were collected. The updated Charlson Comorbidity Index (uCCI) [28], with *International Classification of Diseases 10th Revision* coding algorithms [29], was determined for all patients discharged by triage.

### Data analysis

Continuous data are presented as medians and interquartile ranges (IQR), whereas categorical data are given as numbers and percentages. In the descriptive statistics, the χ2 test was used for ED occupancy level, sex, and triage shifts, while the Mann-Whitney U test was applied for age and uCCI, to evaluate whether there was a significant difference between the revisit and no revisit groups. Furthermore, the predicting variables for revisitation and hospitalization were assessed with univariate and multivariable logistic regression, and the results were presented as odds ratios (OR) with 95% confidence intervals (95% CI). In all analyses, p-values below 0.05 were considered statistically significant.

The ED occupancy level was divided into four groups according to quartiles (Q1–Q4). The least occupied ED quartile (Q1) at the patient’s arrival to triage was considered the reference group for the other quartiles (Q2–Q4). In the further analysis, the occupancy was divided into ten groups according to deciles in order to determine the actual threshold of ED occupancy level that was associated with revisits. The multivariable logistic regression analyses were adjusted for age, sex, uCCI, and triage shift. Age and the uCCI were regarded as continuous variables and sex and triage shift as categorical variables. The triage shift “day” was regarded as the reference. Statistical analyses were performed with IBM SPSS Statistics (Version 29.0.1.0).

## Results

**Fig. 1:**
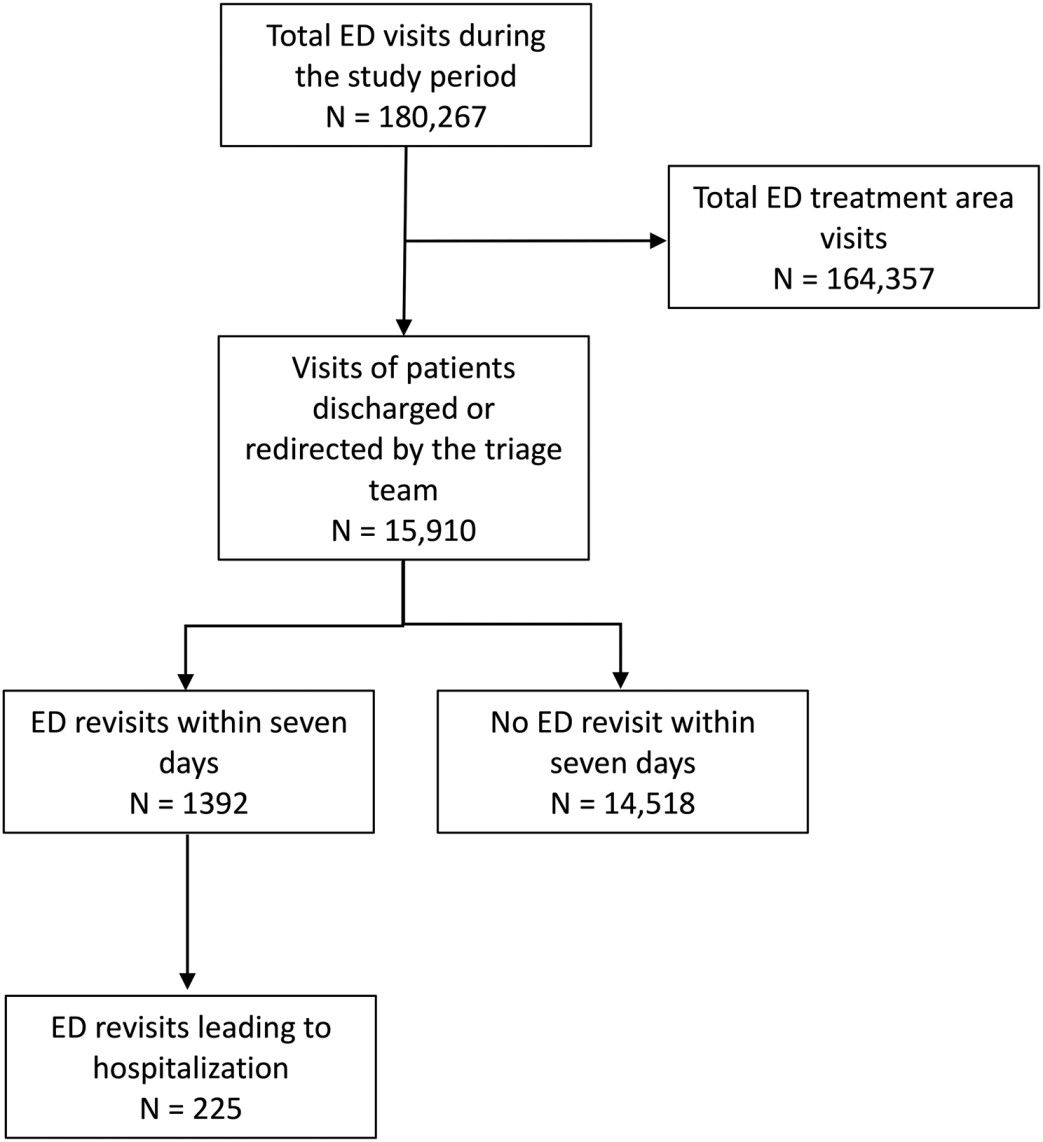
Study selection flowchart. ED, emergency department

During the study period of 2023 and 2024, there were a total of 180,267 ED visits, and 15,910 (8.8%) of these visitors were discharged by triage. Of the discharged patients, 1392 (8.7%) revisited the ED within seven days, and 225 (16.2% of the revisiting patients) were subsequently hospitalized (Fig. 1). The median of ED occupancy during the study period was 54 patients (IQR: 39–67) (Table 1), and the median number of days from triage discharge to revisit was 2 (IQR: 1–4).

**Table 1:** Study population characteristics (*n* = 15,910)

	Characteristics				
	Revisits *N* = 1392		No revisit *N* = 14,518		
	*n* (%) / median [IQR]		*n* (%) / median [IQR]		*p*-value^a^
**ED occupancy at triage discharge**					0.417
Q1 (< 40)	338	(24.3)	3710	(25.6)	
Q2 (40–54)	344	(24.7)	3652	(25.2)	
Q3 (55–67)	351	(25.2)	3688	(25.4)	
Q4 (> 67)	359	(25.8)	3468	(23.9)	
**Basic demographics**					
Male	653	(46.9)	6782	(46.7)	0.888
Age, y	37	[26–60]	31	[22–50]	< 0.001
uCCI score	0	[0–0]	0	[0–0]	< 0.001
0	1064	(76.4)	12,524	(86.3)	
1–3	291	(20.9)	1821	(12.5)	
> 3	37	(2.7)	173	(1.2)	
**ED triage shift**					< 0.001
Day	290	(20.8)	3631	(25.0)	
Evening	679	(48.8)	7163	(49.3)	
Night	423	(30.4)	3724	(25.7)	

ED, emergency department; IQR, interquartile range; uCCI, updated Charlson Comorbidity Index (maximum comorbidity score 24) with ICD-10 coding algorithms

Triage shifts: day 7:30–14:29, evening 14:30–21:59, night 22:00–07:29

^a^The χ2 test was used for all other variables besides age and uCCI, which were analyzed with the Mann-Whitney U test

In the univariate analysis, the rate of revisits within seven days of the initial discharge did not differ significantly between the highest and lowest ED occupancy quartiles. However, after adjusting for confounding factors in the logistic regression analyses, the highest quartile (Q4) of ED occupancy was associated with a revisit within seven days (Table 2). In further analysis, the actual ED occupancy threshold level for an increased probability of revisitation within seven days could not be determined even by dividing the occupancy levels into deciles.

**Table 2:** Factors predicting an ED revisit within seven days (*n* = 15,910)

	Logistic regression					
	Univariate			Multivariable		
	OR	95% CI	*p*-value	OR	95% CI	*p*-value
**ED occupancy at triage discharge**						
Q1 (< 40)	Ref			Ref		
Q2 (40–54)	1.03	0.88–1.21		1.13	0.95–1.33	
Q3 (55–67)	1.05	0.89–1.22		1.17	0.97–1.40	
Q4 (> 67)	1.14	0.97–1.33		1.29	1.06–1.57	0.010
**Basic demographics**						
Male	1.01	0.90–1.13		1.00	0.90–1.12	
Age, for year increase	1.01	1.01–1.02	< 0.001	1.01	1.01–1.02	< 0.001
uCCI, for score increase	1.22	1.17–1.28	< 0.001	1.13	1.07–1.18	< 0.001
**ED triage shift**						
Day	Ref			Ref		
Evening	1.42	1.22–1.66	< 0.001	1.63	1.39–1.91	< 0.001
Night	1.19	1.03–1.37	0.019	1.23	1.03–1.46	0.020

CI, confidence interval; ED, emergency department; OR, odds ratio; uCCI, updated Charlson Comorbidity Index (maximum comorbidity score 24) with ICD-10 coding algorithms

Triage shifts: day 7:30–14:29, evening 14:30–21:59, night 22:00–07:29

The median age was higher in the revisit group than in the no revisit group. Additionally, there were more patients in the revisit group with elevated uCCI scores than in the no revisit group (Table 1). Both univariate and multivariable analyses showed that age and the uCCI score were linked to a higher likelihood of revisitation within seven days after discharge by triage. Furthermore, if the discharge occurred during the evening shift (14:30–21:59) or the night shift (22:00–07:29), the probability of ED revisitation was higher compared to the day shift (7:30–14:29) (Table 2).

In the subgroup analysis of revisits leading to hospitalization, the rate of hospitalization was similar in the highest and lowest occupancy quartiles (Table 3), and neither ED occupancy nor triage shift showed associations with hospitalization. However, older age and a higher uCCI score were associated with the rate of hospitalization in both univariate and multivariable analyses (Table 4).

**Table 3:** Characteristics of hospitalized revisit subgroup (*n* = 15,910)

	Characteristics				
	Hospitalized revisits *N* = 225		No hospitalization *N* = 15,683		
	*n* (%) / median [IQR]		*n* (%) / median [IQR]		*p*-value^a^
**ED occupancy at triage discharge**					0.946
Q1 (< 40)	56	(24.9)	3992	(25.5)	
Q2 (40–54)	55	(24.4)	3941	(25.1)	
Q3 (55–67)	56	(24.9)	3983	(25.4)	
Q4 (> 67)	58	(25.8)	3769	(24.0)	
**Basic demographics**					
Male	107	(47.6)	7328	(46.7)	0.803
Age, y	49	[29–70]	32	[22–50]	< 0.001
uCCI score	0	[0–1]	0	[0–0]	< 0.001
0	152	(67.6)	13,436	(85.7)	
1–3	57	(25.3)	2055	(13.1)	
> 3	16	(7.1)	194	(1.2)	
**ED triage shift**					0.285
Day	65	(28.9)	3856	(24.6)	
Evening	108	(48.0)	7734	(49.3)	
Night	52	(23.1)	4095	(26.1)	

ED, emergency department; IQR, interquartile range; uCCI, updated Charlson Comorbidity Index (maximum comorbidity score 24) with ICD-10 coding algorithms

Triage shifts: day 7:30–14:29, evening 14:30–21:59, night 22:00–07:29

^a^The χ2 test was used for all other variables besides age and uCCI, which were

analyzed with the Mann-Whitney U test

**Table 4:** Factors predicting a revisit leading to hospitalization within seven days (*n* = 15,910)

	Logistic regression					
	Univariate			Multivariable		
	OR	95% CI	*p*-value	OR	95% CI	*p*-value
**ED occupancy at triage discharge**						
Q1 (< 40)	Ref			Ref		
Q2 (40–54)	1.00	0.68–1.45		1.12	0.76–1.66	
Q3 (55–67)	1.00	0.69–1.46		1.19	0.77–1.84	
Q4 (> 67)	1.10	0.76–1.59		1.31	0.82–2.10	
**Basic demographics**						
Male	1.03	0.80–1.35		1.01	0.78–1.32	
Age, for year increase	1.03	1.02–1.03	< 0.001	1.02	1.02–1.03	< 0.001
uCCI, for score increase	1.37	1.27–1.46	< 0.001	1.23	1.13–1.33	< 0.001
**ED triage shift**						
Day	Ref			Ref		
Evening	0.75	0.52–1.09		0.95	0.66–1.39	
Night	0.83	0.61–1.13		0.93	0.63–1.37	

CI, confidence interval; ED, emergency department; OR, odds ratio; uCCI, updated Charlson Comorbidity Index (maximum comorbidity score 24) with ICD-10 coding algorithms

Triage shifts: day 7:30–14:29, evening 14:30–21:59, night 22:00–07:29

## Discussion

The aim of this study was to establish whether an ED’s occupancy level is associated with ED revisits and subsequent hospitalization within seven days after direct discharge by a triage team in a high-volume ED. ED occupancy was used to characterize the crowding status in the ED. The ED features 65 beds, and the highest occupancy quartile (Q4 > 67 patients) thus reflects an ED occupancy ratio (EDOR) of more than one, considering that the study included all ED patients, not just those occupying beds. Over the two-year study period, 8.8% of all ED visitors were discharged or redirected by triage. Of these, 8.7% revisited the ED within seven days, and 16.2% of these revisits resulted in hospitalization. The results are in line with our previous study [30]. However, comparison to other studies is challenging due to the different study settings and follow-up times [8, 9, 12, 21, 24].

According to the results of this study, the highest quartile of ED occupancy at the time of the discharge from triage was associated with an ED revisit within seven days, after adjusting for confounding factors. However, the actual threshold for ED occupancy level that was associated with revisits could not be determined, and higher occupancy was not associated with revisits leading to hospitalization. In an earlier study from the Netherlands, more redirected patients were referred back when the ED was crowded, but the total number of referred patients was considered small [21]. Another study from Sweden found neither decreased permeability of triage at times of high in-hospital bed occupancy nor an association with revisits within 72 h of being redirected to primary care or discharged to go home by triage [24]. While this was not within the scope of the present study, in both previous reports, the higher in-hospital or ED occupancy did not increase the number of redirected patients [21, 24].

An important finding of this study was that older age and a higher uCCI score were linked to increased revisits and subsequent hospitalizations following the initial discharge, confirming the results of our previous study [16]. Although most of the patients in both groups had an uCCI score of 0, a higher uCCI score was associated with revisits and subsequent hospitalization. Therefore, there might be a risk of under-triaging patients with several comorbidities and them being wrongly redirected or incorrectly identified during the triage evaluation. However, the uCCI may not be the most effective tool for indicating ED revisits or subsequent hospitalization, but it provides a widely used practical framework for collecting clinically relevant and comparable comorbidities for research purposes. The association of evening and night shifts with a higher number of revisits can be partly explained with organizational reasons. In the Finnish publicly funded universal healthcare system, the criteria for the interface between emergency and non-emergency care are nationally guided, but the decision on the urgency of care must always be made on a case-by-case basis [27]. Thus, some non-emergent patients were possibly redirected to primary healthcare units during out-of-office hours but later required evaluation or investigations at secondary care hospitals. However, the work shifts were not associated with hospitalization, predicting uniform quality in the performance of triage between shifts.

The results of this study did confirm the hypothesis that ED occupancy affects the triage process, when the number of revisits by discharged patients was used as an indicator. However, patient-related factors, such as demographics, comorbidities, the main complaint during the visit, and other factors [15–18], are more significant predictors of a potential revisit than the temporal ED crowding status at the time of discharge from triage. As was shown in a previous study, in-hospital crowding was unlikely to be associated with reduced permeability in the triage process, when assessing the need for ED care [24]. Little is known, however, about whether ED crowding affects the outcome of redirected or directly discharged patients [1, 21, 24], although the adverse patient outcomes associated with ED crowding have been extensively reported for decades [1]. This is understandable for three main reasons: (1) the effectiveness of redirection strategies in preventing crowding has not been conclusively established, and the strategies are not widely implemented or commonly reported [1, 20]; (2) the definitions for ‘nonurgent’ or ‘inappropriate’ visits vary [13], and there is no unequivocal association between lower triage categories and ‘inappropriate’ ED visits [19, 31]; and (3) the redirection strategies are closely related to the local healthcare systems and legislation.

## Limitations

The present study also has several limitations. This was a single-center retrospective observational study covering a period of two consecutive years. While the data on subsequent ED utilization are comprehensive, the eventual ambulatory utilization of healthcare services remains unknown because there was no regular follow-up for discharged patients or because triage could not specifically redirect patients to a primary care provider. Therefore, it is possible that revisits may have taken place at primary healthcare units or hospitals located outside the Wellbeing Services County of Pirkanmaa. However, Tampere University Hospital is the only hospital in the region managing all severe emergency situations. The distance to the closest hospital with similar resources is 80 km, which justifies the assumption that our data include at least the vast majority of revisits requiring a secondary-care hospital service.

The study included only patients who were discharged by triage. Therefore, the difference in the association of ED occupancy with revisits between directly discharged patients and admitted but discharged walk-in patients who received standard ED care remains unaddressed [20]. Additionally, the study covered all patients discharged by triage during the two-year study period, thus also including planned revisits and revisits for a different reason besides the index visit that originally led to a direct discharge [30]. It is obvious that a longer follow-up time for revisits increases the proportion of revisits that occur for a different reason than the original visit. However, the proportion of revisits after five days was relatively small, circa 11.9% of the revisits included in this study. Furthermore, due to the comprehensive sample size (*n* = 15,910), we were unable to adjust for all previously described potential confounders associated with nonurgent ED utilization, such as demographic and other factors [18] besides age and sex, nor were we able to adjust for all patient- or visit-related factors of the index triage visit [16].

## Conclusions

According to this study, the highest ED occupancy quartile was associated with a modestly increased likelihood of an ED revisit but not hospitalization within seven days after being discharged by the triage team. Furthermore, age and comorbidities were associated with both revisitation and hospitalization. Regardless of the ED crowding status, older patients and patients with comorbidities should receive special attention whenever evaluating the appropriateness of redirecting or directly discharging them from triage.

## Data Availability

The data generated for the current study are not publicly available due to national juridical restrictions protecting sensitive and private information of individual patients in the research data. Further analyses of data are available from the authors upon reasonable request.

## Abbreviations

95% CI: 95% confidence interval
ED: Emergency department
EDOR: Emergency department occupancy ratio
ESI: Emergency severity index
IQR: Interquartile range
OR: Odds ratio
STROBE: Strengthening the Reporting of Observational Studies in Epidemiology
uCCI: Updated Charlson comorbidity index
